# Hybrid Capture-Based Next Generation Sequencing and Its Application to Human Infectious Diseases

**DOI:** 10.3389/fmicb.2018.02924

**Published:** 2018-11-27

**Authors:** Maxime Gaudin, Christelle Desnues

**Affiliations:** IRD 198, CNRS FRE2013, Assistance-Publique des Hôpitaux de Marseille, UMR Microbes, Evolution, Phylogeny and Infections (MEPHI), IHU Méditerranée Infection, Aix-Marseille Université, Marseille, France

**Keywords:** hybrid capture, next generation (deep) sequencing, target enrichment, infectious disease, paleomicrobiology

## Abstract

This review describes target-enrichment approaches followed by next generation sequencing and their recent application to the research and diagnostic field of modern and past infectious human diseases caused by viruses, bacteria, parasites and fungi.

## Introduction

The development of next-generation sequencing (NGS) approaches has revolutionized human clinical research because of its ability to rapidly generate large volumes of sequencing data per run, with a concomitant decrease of sequencing costs (Shendure and Ji, 2008). Unbiased ultra-deep sequencing of complex samples is now accessible, although bioinformatics analyses may still be long and tedious. This issue is particularly problematic in the field of infectious disease diagnostic, where the rapid identification and functional characterization of a particular pathogen is critical for the clinical management of infected patients. So far, polymerase chain reaction (PCR) has been the gold standard method for the clinical diagnosis of infectious diseases (Edwards and Gibbs, 1994). This approach, which is based on the amplification of a generally short and conserved genomic region, can provide information on the presence/absence and abundance of a targeted microbial pathogen. PCR has numerous advantages, such as low cost, rapid processing and results acquisition, automation, sensitivity and specificity. However, and precisely because of its high specificity, PCR may not detect microorganisms whose sequences are too divergent from those targeted by the primers and probes designed. In addition, PCR will provide only partial information on the genetic diversity, genotype, functional potential, nutritional requirements as well as virulence or antibiotic-resistance. Such information, that could only be retrieved from whole genome sequencing (WGS), usually requires culture of the pathogen, which can be unsuccessful in the majority of cases (and particularly for viruses and other intracellular organisms which need host cells), can take several weeks for fastidious microorganisms or can be prevented by early administration of antimicrobial drugs. The power of NGS might thus be of particular interest in that cases for reconstructing full genomes of pathogens directly from nucleic acids extracted from clinical samples. However, due to the low pathogen/nucleic acid ratio in these complex biological samples, NGS may fail to detect/reconstruct genomes from pathogens present in low copy numbers in the sample. To overcome these limitations, capture methods, such as hybridization capture followed by NGS sequencing (also called hybrid-capture sequencing or target-enrichment sequencing) applied directly on human clinical samples have been developed (Mamanova et al., 2010). These approaches allow retrieving large genomic fragments to complete genomes with high sequencing coverage, which facilitate downstream investigations, such as phylogenetics, evolution, epidemiology, and drug resistance. In this review, we will briefly describe the different principles of hybridization capture coupled with NGS, its early developments on human genetic studies and applications in the recent years to the study of present and past human infectious diseases (Gasc et al., 2016) directly from biological samples.

## Overview of the Experimental Procedure and Applications

**FIGURE 1:**
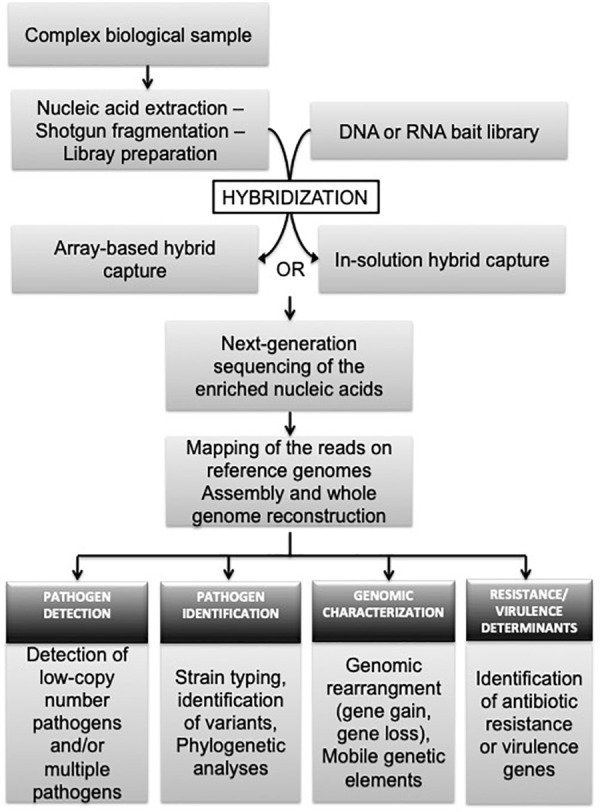
Overview of target-enrichment sequencing procedure and its application to research and diagnostic infectiology.

Next-generation sequencing hybridization-based capture is an approach directly applied after nucleic acid extraction and library preparation (Figure 1). Fragmented shotgun libraries are denatured by heating and subjected to hybridization with DNA or RNA single-stranded oligonucleotides (called also ‘probes’ or ‘baits’) specific to the region of interest (Kozarewa et al., 2015). RNA baits are preferable, because RNA:DNA duplexes are better in term of hybridization efficiency and stability, compared to DNA:DNA hybrids (Lesnik and Freier, 1995). Non-specific unbound molecules are washed away, and the enriched DNA is eluted for NGS (Kozarewa et al., 2015). The hybridization between DNA libraries and baits can be carried out in solution or on a solid support. In “solid-phase,” DNA probes are bound to a solid support, such as a glass microarray slide (Albert et al., 2007; Okou et al., 2007), where in “solution-capture,” free DNA or RNA probes are biotinylated allowing them to isolate the targeted fragment-probe heteroduplexes using magnetic streptavidin beads (Gnirke et al., 2009). So far, there is no standardize protocol for target enrichment processes and several adjustments can be made on library fragment size, sample fragmentation, cleanup procedures, number of PCR amplification cycles, and/or the hybridization duration. Detailed protocols are described in the work of Mamanova et al. (2010) along with values on specificity, sensitivity and reproducibility of each tested procedure. The ultimate step of NGS hybridization-based capture is the sequencing of the enriched nucleic acids and bioinformatic analyses of the reads. The last process usually includes steps of trimming (for adapter sequences, low quality and duplicate reads), mapping of the remaining reads on reference genomes for pathogen detection and identification, and/or assembly into contigs for genome reconstruction (Figure 1). The information provided by the genome can further be explored to investigate the genetic diversity (strain genotyping, variant detection), epidemiology, evolutionary history, transmission networks, and/or antimicrobial resistance of the target pathogen(s).

## Early Developments of Hybrid-Capture Strategies: Human Genetic Studies on Modern and Ancient Samples

Target-enrichment strategy using hybrid capture was originally developed for human genomic studies for which it was used to capture and sequence the entire human exome. This genomic technique, also called exome sequencing (or whole exome sequencing) was first applied by using an array-based hybrid capture method in 2007 (Hodges et al., 2007). In this study, the authors developed six customized NimbleGen arrays to capture about 180,000 coding exons with overlapping 60–90-nt probes allowing an average enrichment of exon DNA sequences of 323 folds. Whole exome sequencing using capture arrays has proven its usefulness in identifying rare variants and mutations causing disease (Choi et al., 2009; Ng et al., 2009). The limitations of this technique include the need to design an array and a relatively large amount of DNA. To overcome some of the weaknesses of the previous method, Gnirke et al. (2009) have developed an in-solution hybrid capture method for human whole exome sequencing. To do so, biotinylated RNA baits of 170 bases in length were constructed, targeting 5,565 human protein-coding exons. In this study, authors have demonstrated the possibility to perform hybrid selection in solution. Following this, many targeted human exome in-solution enrichment methods for NGS have been developed, including those commercialized by Illumina ^1^ and Agilent Genomics ^2^ (Chen et al., 2015a,b). In-solution capture for exome sequencing turned out to be an effective approach applied to discover the causal mutation of rare Mendelian disorders (Shearer et al., 2010; Martignetti et al., 2013; Nectoux et al., 2015; Rousseau-Nepton et al., 2015), of complex disorders (Poultney et al., 2013; Guipponi et al., 2014; Griesi-Oliveira et al., 2015; Pérez-Serra et al., 2015), mitochondrial disorders (Calvo et al., 2012; Gai et al., 2013) and more recently of the screening of potential genetic mutation of patients suffering from cancer (Sikkema-Raddatz et al., 2013; Drilon et al., 2015; Xie et al., 2016; Rozenblum et al., 2017; Xu et al., 2017; Clark T.A. et al., 2018; Schrock et al., 2018).

The power of hybridization capture has been also successfully used to study human ancient DNA (aDNA) preserved in ancient human remains. Indeed, in ancient human samples, DNA is highly fragmented (thus a shotgun fragmentation step is usually not required) and dominated by a large contamination of environmental and bacterial DNA, which poses a limitation in shotgun aDNA sequencing experiment (Knapp and Hofreiter, 2010). Another characteristic of aDNA is cytosine deamination on the ends of DNA fragments. Library construction can be done directly on the double stranded DNAs (dsDNAs) or single stranded DNAs (ssDNAs) and may include cytosine deamination removal by the use of a damage treatment step with uracil DNA glycosylase and/or endonuclease VIII (Briggs et al., 2010). The first genetic marker analyzed in human paleogenetic studies was mitochondrial DNA (mtDNA) because of its higher copy number in the cell than nuclear DNA. Probe hybridization assays used biotinylated DNA or RNA probes targeting the two hypervariable segments of the mtDNA control region (CR) (Briggs et al., 2009; Krause et al., 2010; Maricic et al., 2010; Enk et al., 2013; Kihana et al., 2013; Templeton et al., 2013; Eduardoff et al., 2017; Loreille et al., 2018). Another uniparental marker, the Y-chromosome DNA (Y-DNA), was also used to study aDNA. As each cell possesses only one copy of the Y chromosome, the hybridization capture was carried out to enrich specific genomic regions of the Y chromosome both on solid support (Fu et al., 2013) and in solution (Cruz Dávalos et al., 2017). However, targeting mitochondrial DNA or Y chromosome involves discarding a large proportion of potentially informative sequences present in autosomal DNA. For this reason, Carpenter et al. (2013) reported a new capture-based method, called whole-genome in-solution capture (WISC), using modern DNA as bait covering the entire human genome. This method was applied to 12 ancient human DNA libraries and showed an enrichment of 6 to 159 folds of the sequence mapping to the human genome with enrichments of 2 to 13 folds for unique fragments (Carpenter et al., 2013). As for modern human genetic studies, commercial kits targeting mitochondrial DNA, custom loci, or entire nuclear genomes, such as those developed by Arbor Bioscience (myBaits^®^^3^) are now employed in the genetic sequencing of ancient DNA (Enk et al., 2014; Lindo et al., 2017).

## Applications of Target-Enrichment Sequencing to Human Infectious Diseases

### Parasites and Fungi

The first application of hybrid selection method for infectious diseases was in the field of human parasitology research(Melnikov et al., 2011; Table 1). To overcome the low proportion of *Plasmodium falciparum* sequences relative to that of their human host, authors have proposed to adapt in-solution NGS hybrid capture method to enrich this pathogen. This protocol has been tested in both mock mixtures composed of 99% human DNA and 1% *Plasmodium* but also in *P. falciparum* clinical samples. For this purpose, synthetic 140 bp oligos labeled with biotin were designed to capture exonic regions of the *P. falciparum* genome, whereas 250 bp oligos were constructed to target the entire genome. Processed and unprocessed samples were then sequenced with an Illumina technology. In the mockmetagenome, sequencing of the hybrid-selected samples yielded between 37 to 44-fold enrichment of the parasite DNA. In the human clinical sample, Illumina sequencing showed that at least 5.9% of reads mapped to *Plasmodium*, but no data was provided regarding the percentage of *Plasmodium* reads obtained without hybrid capture (Melnikov et al., 2011). However, this first study highlighted the good performance of NGS hybrid capture to sequence parasite genome from human clinical samples. In 2012, other studies confirmed the good performance of in-solution hybrid capture to enrich *P. falciparum* (Smith et al., 2012) sequences and *P. vivax* (Bright et al., 2012).

**Table 1:** Example of studies that used target-enrichment sequencing for parasitic, fungal, bacterial, or viral diseases in modern and ancient samples.

Targeted organism	Probes design	Infectious disease	Sample tested	Methods	Nucleic acids	Sequencing	Reference
**Parasitic and fungal disease**							

*Plasmodium falciparum 3D7*	Whole genome	Malaria	Mock sample whole blood	In-solution	DNA	Illumina GAIIx Illumina HiSeq	Melnikov et al., 2011
*P. falciparum 3D7*	Whole genome	Malaria	Mock sample Whole blood	In-solution	DNA	Illumina GAIIx	Smith et al., 2012
*Plasmodium vivax*	Whole genome	Malaria	Whole blood	In-solution	DNA	Illumina HiSeq	Bright et al., 2012
*Candida albicans*	6094 ORFS	Systemic candidiasis	Animal model of infection	In-solution	RNA (cDNA)	Illumina HiSeq	Amorim-Vaz et al., 2015

**Bacterial disease**							

*Chlamydia trachomatis*	74 References whole genome	Trachoma	Vaginal swabs Urine	In-solution	DNA	Illumina MiSeq	Christiansen et al., 2014
*Mycobacterium tuberculosis H37Rv*	Whole genome	Multidrug-resistant tuberculosis	Sputum	In-solution	DNA	Illumina MiSeq	Brown et al., 2015
*Neisseria meningitidis*	77 Complete reference genome and 2898 drafts	Invasive meningococcal disease	CSF Whole blood	In-solution	DNA	Illumina MiSeq	Clark S.A. et al., 2018

**Viral disease**							

*Varicella-zoster virus Epstein–Barr virus Kaposi’s Sarcoma-Associated Herpesvirus*	Whole genome	Zoster-vaccine rash, wild-type zoster, encephalitis	Clinical and cultured samples	In-solution	DNA	Illumina GAIIx	Depledge et al., 2011
*Merkel cell polyomavirus (MCPyV)*	23 Overlapping PCR products that tiled across the MCPyV genome	Merkel cell carcinoma	Formalin-Fixed, Paraffin-Embedded Tissue	In-solution	DNA	Illumina GAIIx	Duncavage et al., 2011
*Human Herpesvirus 7 (HHV7)*	Whole genome	Exanthem subitum	Cellular culture	In-solution	DNA	Illumina MiSeq	Donaldson et al., 2013
*Epstein–Barr Virus (EBV)*	Whole genome	Nasopharyngeal carcinoma (NPC)	NPC tumor biopsy	In-solution	DNA	Illumina MiSeq	Kwok et al., 2014
*Zika virus (ZIKV)*	Whole genome	ZIKV infection	Blood, urine, cerebrospinal fluid, and saliva	In-solution	RNA (cDNA)	Illumina HiSeq	Metsky et al., 2017
*Lassa virus*	2 References complete genome	Hemorrhagic fevers	Plasma or serum	In-solution	RNA (cDNA)	Illumina HiSeq	Matranga et al., 2014
*Varicella Zoster Virus (VZV)*	Whole genome	Vaccine-associated rashes	Vesicular fluid	In-solution	DNA	Illumina (GAIIx, HiSeq, and MiSeq)	Depledge et al., 2014
*ViroCap 337 DNA and RNA viral species infecting human and animals*	1456 RefSeq + genome neighbor sequences (185,835 fasta total)	n.a.	Panel of human samples	In-solution	DNA and RNA (cDNA)	Illumina HiSeq	Wylie et al., 2015
*VirCapSeq-VERT 207 viral taxa infecting vertebrates*	342,438 Coding sequences (1,993,200 oligonucleotide probes)	n.a.	Mock samples Human clinical samples and animal samples	In-solution	DNA and RNA (cDNA)	Illumina HiSeq	Briese et al., 2015
*Norovirus*	662 References complete or partial genome	Acute gastroenteritis	Stool suspensions	In-solution	RNA (cDNA)	Illumina MiSeq	Brown et al., 2016
*Hepatitis C Virus (HCV)*	953 Reference complete genomes	HCV infections	Plasma Mock samples RNA transcripts	In-solution	RNA (cDNA)	Illumina MiSeq	Thomson et al., 2016
*Exogenous retroviruses* Immunodeficiency virus type-1 (HIV-1) and the human T-cell leukemia virus type-1 (HTLV-1)	Whole genome	n.a.	Infected cells	In-solution	DNA	Illumina MiSeq	Miyazato et al., 2016
*Herpes simplex virus 1 and 2 (HSV-1/HSV-2)*	Whole genome	Skin rash, herpetic lesions	Swab of the lesion	In-solution	DNA	Illumina MiSeq	Greninger et al., 2018
*ViroFind 535 DNA and RNA viral species that are known to infect humans*	Whole genome	Progressive multifocal leukoencephalopathy (PML)	^:^Post mortem PML brain samples	In-solution	DNA and RNA (cDNA)	Illumina MiSeq	Chalkias et al., 2018

**Infectious diseases in paleomicrobiology**							

*Yersinia pestis strain CO92*	Whole genome or pCD1 and pMT1 plasmids	Plague (Black Death) 1347–1351	Teeth	Microarray	DNA	Illumina GAIIx	Bos et al., 2011
*Y. pestis strain CO92*	Portion of the *Y. pestis* pPCP1 plasmid	Plague (Black Death) 1347–1351	Bones and Teeth	In-solution	DNA	Illumina GAIIx	Schuenemann et al., 2011
*Y. pestis strain CO92*	Core genome, plasmids and 155 other genes	Plague (Justinian) (6–8th centuries)	Teeth	In-solution	DNA	Illumina HiSeq	Wagner et al., 2014
*Mycobacterium tuberculosis*	rpoB, gyrA, gyrB, katG, and mpt40 genes	Tuberculosis (1000 year old)	Skeletal samples	In-solution	DNA	Illumina HiSeq	Bos et al., 2014
*Mycobacterium leprae*	3 Genomic loci	Leprosy (11th to 14th century)	Bones and teeth	In-solution	DNA	Illumina MiSeq and HiSeq	Schuenemann et al., 2013
*Variola virus*	Whole genome	Smallpox (1643–1665)	Mummified tissues of a child	In-solution	DNA	Illumina HiSeq	Duggan et al., 2016
*P. falciparum*	Mitochondrial genomes of *Plasmodium* spp.	Malaria (1st–2nd century)	Teeth	In-solution	DNA	Illumina HiSeq	Marciniak et al., 2016
*Treponema pallidum*	Whole genome	Syphilis (1681 to 1861)	Bones	Microarray	DNA	Illumina HiSeq	Schuenemann et al., 2018

*n.a., not applicable.*

Fungi are also a major cause of human diseases that can be particularly serious in immunocompromized patients or in patients hospitalized for serious diseases (Pfaller and Diekema, 2010). For example, systemic infections with *Candida albicans* in immunocompromized patients result in mortality rates of about 50% (Pfaller and Diekema, 2010). The prevention, diagnosis and therapy of fungi infections remain very difficult and comprehension of transcriptional regulation between fungal pathogens and host is an important step to identify potential novel targets for drug development (Pfaller and Diekema, 2010). Again, the limitations of host and pathogen transcriptome analysis lie in the low proportion of fungal RNA present in the total extracted RNA. The use of specific enrichment procedures before RNASeq analysis has then been proposed as an alternative method to overcome the problem of low fungus/host RNA ratio. For this purpose, Amorim-Vaz et al. (2015) have designed a set of 55,342 biotinylated 120 bp-RNA probes covering 6,094 *C. albicans* ORFs. cDNA libraries were established using SureSelect (Agilent) after extraction of RNA from mice kidney or *Galleria mellonella* larvae infected with *C. albicans*, and were subjected to capture with biotinylated probes before Illumina HiSeq sequencing. Results showed up to a 1670-fold enrichment of *C. albicans* reads in a given biological sample and a detection of more than 86% of its genes. Many genes that have been regulated in *in vivo* infection experiments have functions that have not yet been characterized and will require further research to understand their role during infection (Amorim-Vaz et al., 2015).

### Bacteria

In bacteriological research and diagnostic, targeted capture strategies prior to sequencing could be a powerful tool in the management and therapeutics of patients with infectious disease. Indeed, the rapid identification of antimicrobial resistance is essential for a rapid and effective treatment. Regarding *Mycobacterium tuberculosis*, current methods of screening for antimicrobial resistance, which are based on the culture of the organism from sputum samples before sequencing, can take up to several weeks. To overcome these limitations, Brown et al. have proposed to use oligonucleotide enrichment technology to capture *M. tuberculosis* genome sequences directly from positive smear sputum samples (Brown et al., 2015; Table 1). Whole genome baits (120-mer RNA baits) were designed to span the entire positive strand of the *H37Rv M. tuberculosis* reference genome and synthesized by Agilent Technologies. The authors demonstrated the reliability of targeted sequencing to recover and sequence, in less than 96 h, nearly complete genomes directly from 81% (21/26) smear positive sputa but also its robustness to identify the genotype and resistance determinants of all samples that were previously tested positive samples. This study emphasizes the use of hybrid selection target enrichment that could allow personalized antimicrobial treatment in multidrug-resistant tuberculosis (Brown et al., 2015). Other studies have used biotinylated baits spanning entire genomes for high-resolution strain genotyping directly from clinical samples. Indeed, discrimination of *Chlamydia trachomatis* serovars from genital samples would facilitate the study of population structures and modes of transmission (Christiansen et al., 2014) while genomic data from uncultured *Neisseria meningitidis* not grown in the case of invasive meningococcal would allow increased surveillance of vaccine antigens and studies on possible vaccine deficiencies (Clark S.A. et al., 2018).

### Viruses

In viral research and diagnostic laboratories, viral WGS is also essential for the detection of drug resistance and the development of novel treatments and vaccines (Houldcroft et al., 2017). In this domain, the first study that demonstrated the effectiveness of target capture technology for reconstructing full herpesvirus genomes from complex biological samples was proposed by Depledge et al. (2011) (Table 1). In this study, 120-mer RNA baits generating a 2× coverage for Varicella-Zoster Virus (VZV), a 5× coverage for Epstein-Barr virus (EBV) and Kaposi’s sarcoma-associated Herpesvirus (KSHV), were synthetized and hybridized with DNA extracted from a range of clinical samples including blood, saliva, vesicle fluid, cerebrospinal fluid, and tumor cell lines. Full-length herpes virus genomes were reconstructed at high read depth for the 13 samples tested and generated further studies on the structure and diversity of the viral population (Depledge et al., 2011). Following this study, the capture of whole genomic hybrids made it possible to study the genomic diversity of eight new complete EBV genomes isolated from biopsy specimens of primary nasopharyngeal carcinomas (Kwok et al., 2014), 37 Zika virus genomes (ZIKV) samples out of 66 attempts (Metsky et al., 2017), 453 complete genomes (with >90% genome coverage and >100-fold read depth) of different norovirus genotypes from 509 stool samples (Brown et al., 2016) and to achieve sufficient coverage for *de novo* genome assembly and detection of single nucleotide variants of Lassa virus (LASV) from ultra-low input samples (Matranga et al., 2014). This approach has been also used to characterize other clinically relevant viruses, such as hepatitis C virus (HCV) (Thomson et al., 2016), varicella zoster virus (Depledge et al., 2014), human herpesvirus 7 (HHV-7) (Donaldson et al., 2013, 7) and the herpes simplex virus 1 and 2 (HSV-1 and HSV-2) (Greninger et al., 2018). Hybrid capture associated with shotgun sequencing could also be performed using a combination of several viral species used as baits. Indeed, Wylie et al. (2015) developed ViroCap, a panel of probes designed to enrich nucleic acid from 34 families of DNA and RNA viruses (190 viral genera and 337 species) that infect vertebrate hosts, except human endogenous retrovirus. These probes were tested both on a pool of 14 clinical samples, which tested positive for a viral infection, and on eight samples from young children with fever, also positive for one or more viruses. Libraries were sequenced before capture (pre-capture) and following capture using ViroCap (post-capture). Combining results from both experiments, 32 viruses were detected (11 additional in the post-captured samples), including diverse DNA and RNA viruses (with genomes ranging from 5–161 kb) with genomic coverage >80% for 16 of the 32 genomes. Several complete genomes were reconstructed and belonged to Human bocavirus 1, Human parvovirus B19, human adenovirus B (type 3), human adenovirus C (type 1), KI polyomavirus, sapovirus, and human astrovirus 1. Finally, although ViroCap cannot detect viral sequences that are completely novel, its design, which includes neighbor genomes of reference sequences, allows variants with nucleotide sequence identity as low as 58% to be identified (Wylie et al., 2015). The same year, VirCapSeq-VERT, a virome capture sequencing platform targeting 207 viral taxa infecting vertebrates was described (Briese et al., 2015). VirCapSeq-VERT allowed reduction of background human DNA and a 100 to 10,000 fold enrichment in viral reads when compared with other enrichment procedures such as treatment with nucleases or RiboZero rRNA depletion (Briese et al., 2015). In 2018, a similar approach called ViroFind was designed to target 535 DNA and RNA viruses, which are known to infect humans or cause zoonoses. This in-solution target enrichment was applied to the brain biopsy samples of five patients with progressive multifocal leukoencephalopathy (PML) (Chalkias et al., 2018). It allowed the description of highly complex Polyoma virus JC populations as well as the detection of large genetic divergence among variants, with some of these mutations conferring viral fitness advantages (Chalkias et al., 2018). Lastly, other applications of target-enrichment sequencing have been described, such as the study of viral genome integrations within the human genome. This approach was powerful and efficient to identify Merkel Cell Polyomavirus (MCPyV) insertion sites on DNA extracted from formalin-fixed and paraffin-embedded tissue from Merkel cell carcinoma (Duncavage et al., 2011). It also allowed to analyze retroviral genomes integrated within host genomic DNA in case of human T-cell leukemia virus type-1 (HTLV-1) and human immunodeficiency virus type-1 (HIV-1) infections (Miyazato et al., 2016).

### Paleomicrobiology

Paleomicrobiology is an emerging research field dedicated to the detection, identification and characterization of microorganisms (bacteria, viruses, and parasites) in ancient specimens. Elucidating past infectious diseases can lead to a better understanding of the temporal and geographical distributions of infected individuals, the introduction of microorganisms into human populations, the host-pathogen relationships but also the genetic evolution of the microorganisms (Drancourt and Raoult, 2005). The main limitations of paleomicrobiological studies concern the degradation of ancient DNA (aDNA) and the risk of contamination by modern DNA (Riviera-Perez et al., 2016). Target-enrichment prior to sequencing is therefore a particularly relevant tool in this context for genomes study. The first two studies using targeted enrichment in paleomicrobiology have investigated genetic changes and virulence factor of *Yersinia pestis*, the causal agent of the second plague pandemics (Black Death, 14–17th centuries) (Bos et al., 2011; Schuenemann et al., 2011; Table 1). To this end, an array-based enrichment using probe targeting either the full *Y. pestis* chromosome or *pestis*-specific virulence plasmids was applied directly after the DNA extraction from ancient bones (Schuenemann et al., 2011, 1) and/or teeth (Bos et al., 2011; Schuenemann et al., 2011). Using targeted DNA capture approach combined with high-throughput sequencing, the authors reconstructed 99% of the pPCP1 plasmid sequence (Schuenemann et al., 2011) and a draft genome of *Y. pestis* (Bos et al., 2011) with the molecular damages typically associated with aDNA. Comparisons with modern genomes did not identify any significant genetic variation that could explain the differences between the ancient and modern forms of the disease (Schuenemann et al., 2011). More recently, three other draft genomes of *Y. pestis* have been recovered from individuals who died during the first plague pandemics (the Plague of Justinian, 6–8th centuries) in two different rural sites in southern Germany (Wagner et al., 2014; Feldman et al., 2016). Genetic characterization showed that these 3 drafts derived from a single Justinianic strain which is unique and harbors novel substitutions and structural polymorphism (Wagner et al., 2014; Feldman et al., 2016). Finally, target enrichment sequencing also allowed the reconstruction of new *Y. pestis* strains from Bronze Age individuals (∼3,800 BP) (Spyrou et al., 2018) providing further datas into the early stages of *Y. pestis* genome evolution including on genomic characteristics supporting flea-borne transmission in rodents or humans (Spyrou et al., 2018). Finally, target-enrichment sequencing approaches in the paleomicrobiological research field have not been exclusively applied to the study of ancient plague pandemics, but have also allowed genomic investigation of ancient *Mycobacterium tuberculosis* (Bos et al., 2014), *M. leprae* (Schuenemann et al., 2013), *Variola virus* (Duggan et al., 2016), *P. falciparum* (Marciniak et al., 2016), and *Treponema pallidum* (Schuenemann et al., 2018) in human remains.

## Conclusion

Target-enrichment sequencing is an efficient approach that allows large fragments and even entire sequences of the genome of targeted microorganisms to be reconstructed directly from modern and ancient complex biological samples containing a low pathogen/host nucleic acid ratio. The information provided by the genome can be used to explore the genetic diversity, epidemiology, evolutionary traits, transmission networks, host-pathogen interactions or antimicrobial resistance of the target pathogen or its variants. The main current limitations of democratizing target-enrichment sequencing in clinical diagnostic laboratories are its elevated cost, the high expertise required for library preparation and the necessary time to generate biotinylated probes from reference genomes, which hampers a rapid response to an emerging pathogen. Above all, it is not suitable for the detection and characterization of completely novel microorganisms, including viruses whose emergence may represent one of the main threats to human health in the near future.

## Author Contributions

All authors listed have made a substantial, direct and intellectual contribution to the work, and approved it for publication.

## Conflict of Interest Statement

The authors declare that the research was conducted in the absence of any commercial or financial relationships that could be construed as a potential conflict of interest.

## Footnotes

1 www.illumina.com

2 www.genomics.agilent.com

3 http://www.arborbiosci.com
